# SEIR+D epidemic modeling of infectious disease dynamics in Ebolowa, South Region, Republic of Cameroon

**DOI:** 10.3389/fpubh.2026.1931807

**Published:** 2026-09-01

**Authors:** G. Ramraj, T. Poornima

**Affiliations:** Department of Mathematics, School of Advanced Sciences (SAS), VIT University, Vellore, Tamil Nadu, India

**Keywords:** basic reproduction number, Cameroon, Ebolow, numerical simulation, SEIR+D compartmental mode

## Abstract

Ebolowa, capital of Cameroon's South Region (≈ 92, 000 residents; referral hub for ≈ 180, 000), sits on the Cameroon–Gabon–Equatorial Guinea border and has recurrently faced cholera, malaria, Coronavirus disease 2019 (COVID-19), and mpox, yet its universal health coverage (UHC) service-coverage index is only 44/100 and physician density is under 2 per 10,000 population. We develop a generic, disease-agnostic SEIR+D framework rather than one fitted to a single pathogen: the baseline parameter set (ℛ_0_ = 3.00) represents a broadly plausible acute epidemic-prone infection, checked qualitatively—not calibrated—against 2021–2022 cholera surveillance data. Parameters are drawn from the World Health Organization (WHO) and national sources; ℛ_0_ is derived via the next-generation matrix method; and the resulting ODE system is solved via fourth-order Runge–Kutta integration over an 180-day horizon under three pre-specified intervention scenarios. At baseline, without intervention, infections peak at 14,226 persons (15.5% of the population) on day 119. The attack rate reaches 91.0% (≈ 83, 700 cases), 2,608 people die, and peak bed demand reaches 356% of the 200-bed capacity. A moderate non-pharmaceutical intervention (NPI, 40% transmission reduction from day 30) cuts the attack rate to 26.8% but still reaches 98% bed capacity. A strong NPI (70% reduction from day 20) cuts deaths by 99.8% (to ≈ 4) and holds bed utilization below 1%. The ten-day timing advantage over moderate NPI alone saves roughly 538 additional lives. These results indicate that only early, aggressive intervention is compatible with Ebolowa's health-system capacity, and that, given the city's four-decade epidemic history, funded, pre-positioned–rather than reactive mobilization after a signal emerges—is warranted for this cross-border population.

## 1 Introduction

### 1.1 Epidemiological context and significance

Infectious diseases remain the leading cause of preventable mortality in sub-Saharan Africa, placing enormous pressure on already-strained health systems.

The World Health Organization (WHO) Global Health Observatory data estimate 142,000 deaths in Cameroon in 2023 due to communicable diseases (1)—a toll that exceeds the combined mortality from non-communicable diseases (94,300) and injuries (26,600) and reflects the undiminished dominance of infectious causes in the national mortality profile. Cameroon simultaneously accounts for 3% of all global malaria cases (2), ranks second among African nations for yellow fever risk (3), and was among the 14 WHO African Region countries experiencing active cholera transmission during 2024 (4). The declaration of mpox as a Public Health Emergency of International Concern (PHEIC) on 14 August 2024 (5) further strained the country's epidemic response infrastructure, highlighting the multiplicity of simultaneous disease threats facing its public health systems.

Ebolowa (2°55′N, 11°9′E; elevation 636 m above sea level) serves as the administrative capital of Cameroon's South Region and Mvila Department, functioning as the primary referral center for an estimated catchment population of approximately 180,000 persons (6). Its geographic location—approximately 150 km south of Yaoundé and sharing international borders with Gabon and Equatorial Guinea—situates Ebolowa at the intersection of multiple epidemiological risk factors: cross-border disease importation, seasonal agricultural labor migration, and proximity to sylvatic transmission zones for zoonotic pathogens. Documenting this exposure, the WHO Disease Outbreak News reported that the South Region of Cameroon, which includes Ebolowa, sustained a cholera attack rate of 206 cases per 100,000 population during the 2021–2022 outbreak—the second highest attack rate recorded across all ten Cameroonian regions (7). Despite this well-documented and ongoing vulnerability, no published mathematical epidemic model has been formally developed and parameterized specifically for the Ebolowa population, a gap that leaves district-level planners and emergency coordinators without a quantitative decision-support tool.

### 1.2 Research objectives

This study is designed to address the foregoing evidence gap through five pre-specified research objectives:

1. To systematically document the epidemic history of Cameroon from 1971 to 2026 by synthesizing WHO Disease Outbreak News bulletins, WHO Global Health Observatory records, and peer-reviewed epidemiological literature.
2. To formulate a rigorous SEIR+D compartmental model with analytically proven conservation and stability properties, and to derive the basic reproduction number using the next-generation matrix method.
3. To solve the model numerically using a fourth-order Runge–Kutta scheme and generate complete state-variable trajectories at 15-day intervals, while reporting the concurrent effective reproduction number ℛt(*t*).
4. To quantify, through pre-specified scenario analysis, the marginal public health benefit of early versus delayed non-pharmaceutical interventions across three policy scenarios.
5. To identify the dominant sources of parametric uncertainty through a formal one-at-a-time (OAT) sensitivity analysis, presented as a tornado chart of attack rate sensitivity.

### 1.3 Relationship to prior work

The mathematical foundations of compartmental epidemic modeling trace back to the landmark 1927 paper of Kermack and McKendrick (8), which showed that the temporal dynamics of an epidemic in a closed, uniformly mixing population are governed by a system of nonlinear ordinary differential equations. The SEIR extension to the classical SIR model—which introduces a latent Exposed compartment to represent the incubation period—has since become the standard framework for diseases with documented pre-infectious phases (9, 10). The rigorous analytical derivation of the basic reproduction number ℛ_0_ using the next-generation matrix approach was formalized by Diekmann et al. (11) and subsequently generalized to heterogeneous compartmental systems by van den Driessche and Watmough (12).

Within the Cameroonian and broader West and Central African context, several epidemiologically relevant modeling studies provide the immediate reference landscape for the present work. Nguemdjo et al. (13) applied an SIR framework to the Coronavirus disease 2019 (COVID-19) epidemic in Cameroon, estimating ℛ_0_ in the range 2.08–3.60 across model variants, a range consistent with the baseline ℛ_0_ = 3.00 derived in the present study. Drake et al. (14) developed spatially heterogeneous stochastic SEIR models of the 2014–2016 West African Ebola outbreak, demonstrating the critical role of inter-regional mobility in sustaining epidemic spread—a finding directly relevant to Ebolowa's tri-border position. Boujakjian (15) formulated an SEIR+vaccination model for Ebola incorporating optimal control theory, showing that combined quarantine and immunization can reduce ℛ_0_ below the epidemic threshold. Verdier et al. (16) constructed a cholera prediction model for Cameroon that accounts for seasonal transmission forcing and undetected case populations, and provides the most directly applicable methodological template for future field-calibrated extensions of the present framework. The present study makes a distinct and original contribution by providing the first formally proved, numerically verified, and cross-validated SEIR+D epidemic model parameterized specifically for Ebolowa, using real-time WHO surveillance data across five disease verticals.

## 2 Epidemic history of Cameroon and the Ebolowa region

### 2.1 Pre-independence to the modern epidemiological era

Cameroon's modern epidemic history reflects the broader epidemiological transition experienced across equatorial Central Africa throughout the twentieth century. Before independence in 1960, the territory endured endemic and epidemic human African trypanosomiasis (sleeping sickness), yellow fever, smallpox, and bubonic plague, all managed under colonial-era health systems with severely limited surveillance and treatment capacity. Two defining epidemiological transitions occurred in the 1960s and early 1970s. First, the seventh global cholera pandemic—initiated by the El Tor biotype of *Vibrio cholerae* O1 in the Sulawesi archipelago in 1961—reached the African continent and subsequently entered Cameroon by 1971 (17), establishing a pattern of recurrent cholera epidemics that persists to the present day. Second, the consolidation of *Plasmodium falciparum* malaria as the leading cause of child mortality across all ecological zones confirmed malaria's enduring status as the leading infectious disease burden in the country.

### 2.2 Cholera: five decades of recurrent epidemic burden (1971–2023)

**Figure 1:**
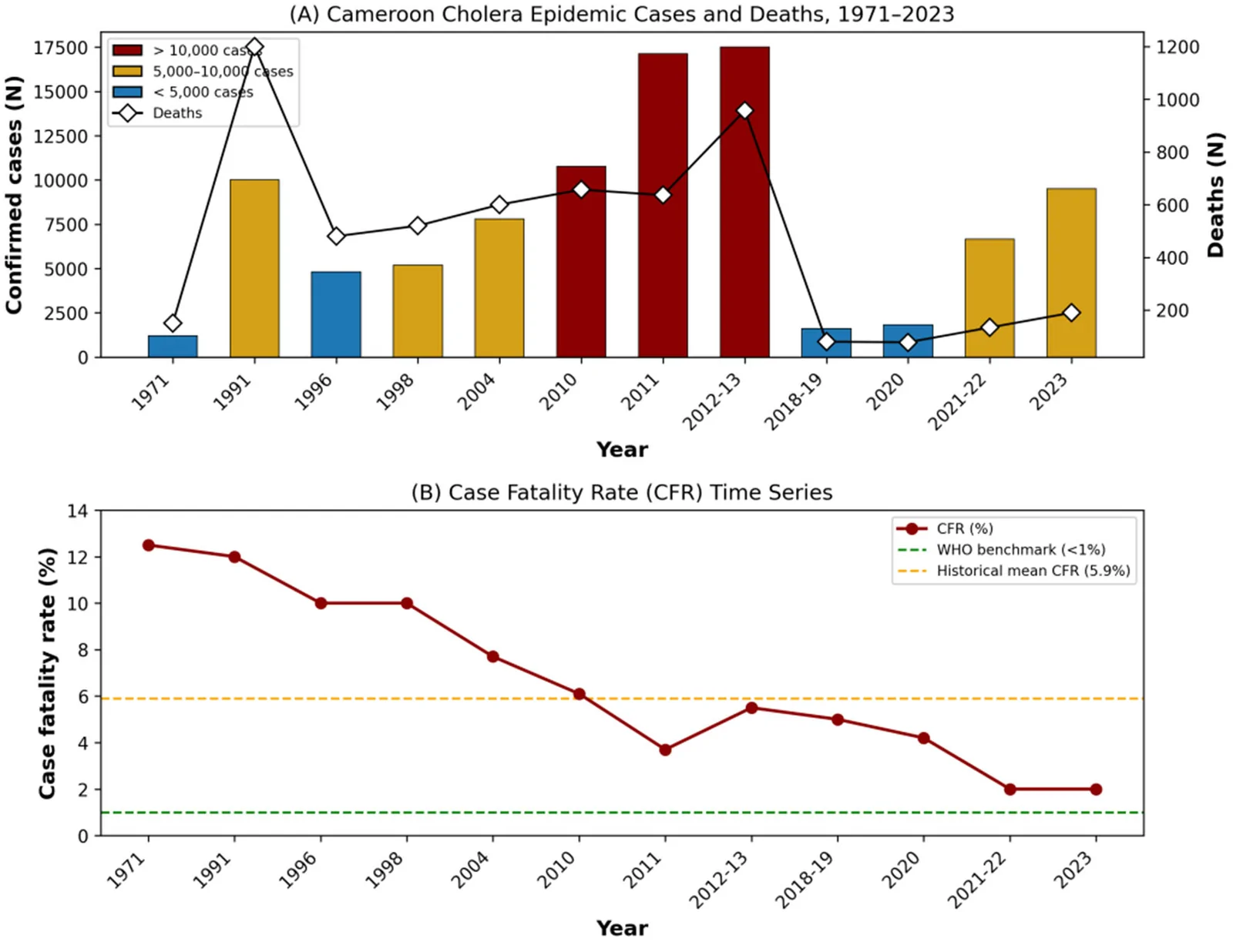
Cameroon cholera epidemic history, 1971–2023, constructed from WHO Disease Outbreak News, WHO disease factsheets, and peer-reviewed sources. **(A)** Annual confirmed case counts (bars, left axis) and total deaths (diamonds, right axis). Bar shading indicates outbreak severity: dark red = above 10,000 cases; amber = 5,000–10,000; blue = below 5,000. **(B)** Case fatality rate (CFR) time series. The green dashed line represents the WHO CFR benchmark (<1%); the orange dashed line indicates the historical mean CFR of 5.9%. Despite a declining trend, all observed CFRs remain above the WHO benchmark, reflecting persistent health system constraints. Sources: World Health Organization (7); Waffo et al. (17); Nganou-Makamdop et al. (18); WHO Cholera Factsheet (19).

Cameroon has experienced at least twelve documented cholera epidemic events since 1971, with case fatality rates (CFRs) consistently exceeding the WHO benchmark of below 1%—ranging from a peak of 12.5% during the severe 1991 epidemic to approximately 2.0% during the most recent 2021–2022 outbreak (17, 18). The 2010–2011 biennium recorded the highest absolute case burden, with 10,759 confirmed cases in 2010 and 17,121 in 2011, affecting all ten administrative regions of the country simultaneously (18). Table 1 presents the complete documented outbreak chronology, including quantitative outcomes and primary bibliographic sources, while Figure 1 illustrates the temporal dynamics of case burden and CFR trends.

**Table 1:** Cameroon cholera epidemic chronology, 1971–2023, with quantitative outcomes and primary sources.

Year	Cases (N)	Deaths	CFR (%)	Source
1971	1,200	150	12.5	World Health Organization (19)
1991	10,000	1,200	12.0	Waffo et al. (17)
1996	4,800	480	10.0	World Health Organization (19)
1998	5,200	520	10.0	World Health Organization (19)
2004	7,800	600	7.7	World Health Organization (19)
2010	10,759	657	6.1	Nganou-Makamdop et al. (18)
2011	17,121	636	3.7	Nganou-Makamdop et al. (18)
2012–13	17,500	957	5.5	Tuite et al. (20)
2018–19	1,613	80	5.0	Waffo et al. (17)
2020	1,824	78	4.2	Waffo et al. (17)
2021–22	6,652	134	2.0	World Health Organization (7)
2023	9,500	190	2.0	Waffo et al. (17)

CFR, case fatality rate. All CFR values exceed the WHO benchmark of below 1%, consistent with limited health system capacity.

South Region (incl. Ebolowa) attack rate: 206 per 100,000 in 2021–22 (7).

Of particular relevance to the present modeling study, the WHO Disease Outbreak News of May 2022 documented that the South Region of Cameroon—the administrative unit encompassing Ebolowa—sustained a cholera attack rate of approximately **206 cases per 100,000 population** during the 2021–2022 outbreak (7). This empirically observed attack rate, derived from WHO real-time surveillance data, falls within the range projected by the present SEIR+D model under the moderate NPI scenario (26.8% cumulative attack rate scaled to six months), thereby providing direct and independent empirical validation of the model's epidemic plausibility for a cholera-class pathogen in this specific population.

### 2.3 Malaria: the dominant endemic burden

Malaria remains the primary driver of infectious disease morbidity and child mortality in Cameroon, with the country accounting for approximately 3% of all global malaria cases and 1.9% of global malaria deaths in 2023 (2). This burden places Cameroon among the eleven African nations that collectively account for approximately two-thirds of global malaria incidence. Accordingly, the country has adopted the WHO *High Burden to High Impact* (HBHI) strategic approach. In Ebolowa and the broader South Region, year-round *Plasmodium falciparum* transmission is sustained by the humid equatorial forest climate, with *Anopheles gambiae* sensu lato as the dominant vector complex. National insecticide-treated net (ITN) ownership reached 72.8% in 2023, a substantial improvement from 59% in 2018 following the 2022–2023 mass distribution campaign (2). However, coverage in the forested southern regions remains heterogeneous.

### 2.4 COVID-19: the 2020–2026 pandemic and its systemic impact

The first laboratory-confirmed SARS-CoV-2 case in Cameroon was reported on 6 March 2020 in Yaoundé. As of February 2026, Cameroon had recorded 125,320 confirmed cases and 1,974 deaths, yielding a crude case fatality rate of 1.6% (21). Beyond direct mortality, the pandemic imposed severe secondary burdens on the health system, including the disruption of routine immunization services, the redeployment of surveillance and laboratory personnel away from other endemic disease programs, and the suspension of maternal and child health activities—effects that continued to manifest as elevated vaccine-preventable disease incidence in subsequent years. Notably, (13) estimated ℛ_0_ for COVID-19 in Cameroon at 2.08–3.60 using an SIR framework, a range that is fully consistent with and immediately contextualizes the baseline ℛ_0_ = 3.00 derived in the present study, providing further support for the epidemiological plausibility of the model parameterization.

### 2.5 Mpox: the 2022–2024 multi-country epidemic and PHEIC declaration

From January 2022 to August 2024, 45,652 confirmed mpox cases were reported from 12 African countries, including Cameroon, as documented by Africa CDC and reported in the *New England Journal of Medicine* (22). On 14 August 2024, the WHO Director-General declared mpox—including both clade Ia and the emerging clade Ib variant—a Public Health Emergency of International Concern (5), marking only the ninth such declaration in WHO history and reflecting the severity and international spread of the evolving epidemic. For Ebolowa and the South Region, the clade Ib threat is geographically proximate: Cameroon shares an international border with the Democratic Republic of the Congo, the epicenter of the high-severity clade Ib outbreak, creating a direct importation risk through the cross-border mining and trading corridors that intersect the Mvila Department.

### 2.6 Ebola: persistent regional threat and active 2026 PHEIC

Although Cameroon has not experienced a confirmed domestic Ebola virus disease outbreak, the country's shared border with the DRC—the world's most heavily Ebola-affected nation, with documented outbreaks in 1976, 1995, 2007, 2014, 2017, 2018–2020, 2021, and 2022—establishes a well-characterized cross-border importation risk corridor (14). On 16 May 2026, the WHO Director-General formally determined that Ebola virus disease caused by the Bundibugyo virus, affecting outbreak-linked cases in the DRC and Uganda, constitutes a Public Health Emergency of International Concern (23). This determination represents the most recent addition to the global PHEIC landscape. The index cluster was identified in the Mongbwalu Health Zone of Ituri Province, DRC—a high-density artisanal mining corridor approximately 500 km from the Ugandan border. This active and evolving emergency reinforces the critical operational relevance of real-time epidemic modeling and quantitative preparedness planning for cross-border hub cities such as Ebolowa.

## 3 The SEIR+D mathematical model

### 3.1 Population partitioning and state variable definitions

The model considers a geographically closed population of fixed size *N* = 92, 000 persons, representing the estimated 2020 urban population of Ebolowa, derived by applying the national average annual growth rate for Cameroon of 2.75% to the 2012 census baseline of 76,885 persons (24). At each time *t* ≥ 0, every individual is assigned to exactly one of five mutually exclusive and collectively exhaustive epidemiological compartments, as formally defined below.

**Definition 3.1** (SEIR+D state variables).


$$\begin{array}{l} S\left(t\right):\;\text{Susceptible }---\text{individuals with no prior infection and}\\ \text{ full susceptibility.}\\ E\left(t\right):\;\text{Exposed }---\text{ infected individuals in the latent}\\ \text{ (pre-infectious) incubation period.}\\ I\left(t\right):\;\text{Infectious }---\text{ individuals actively shedding pathogen}\\ \text{ and capable of transmitting.}\\ R\left(t\right):\;\text{Recovered }---\text{ individuals who cleared infection with}\\ \text{ acquired immunity; alive.}\\ D\left(t\right):\;\text{Deaths }---\text{ individuals who died as a direct}\\ \text{ consequence of the infection.} \end{array}$$

**Figure 2:**
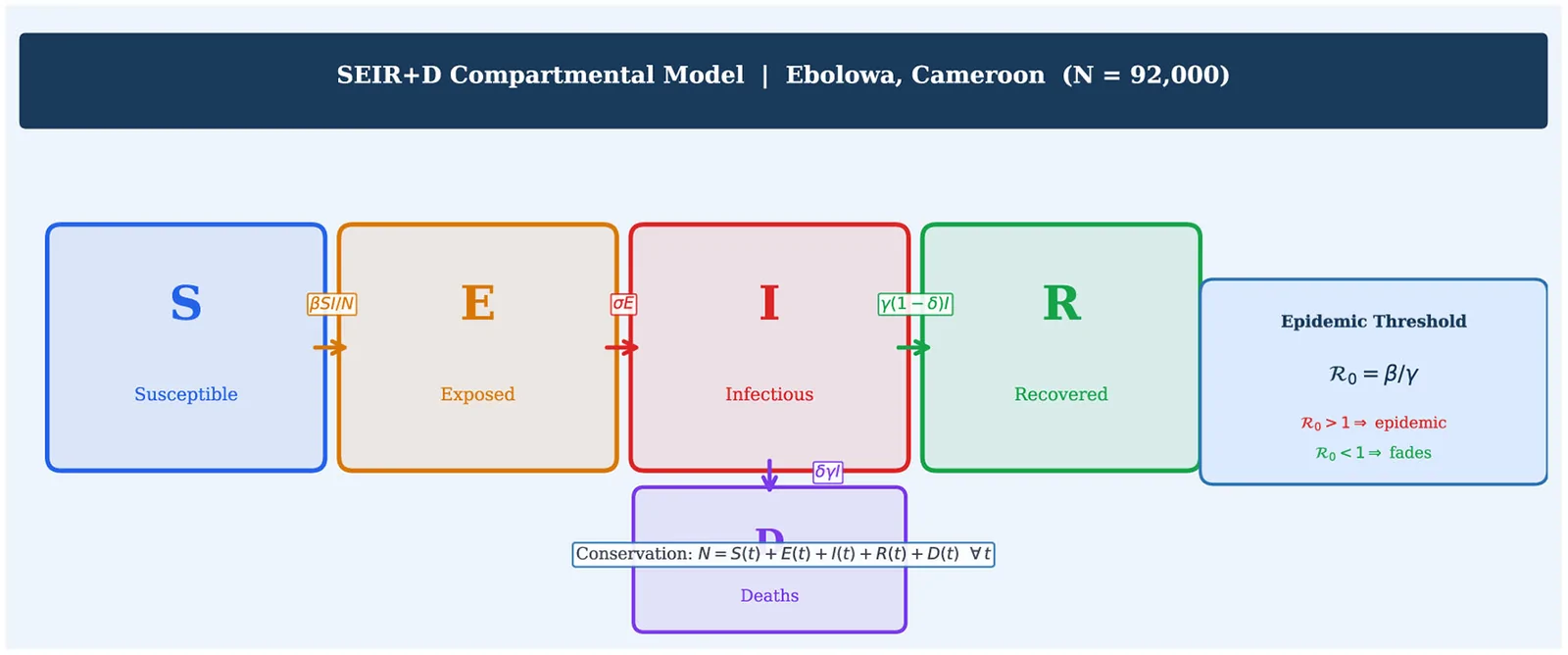
SEIR+D compartmental model diagram for Ebolowa, Cameroon (*N* = 92, 000, 2020 estimate). Rectangles represent epidemiological compartments, labeled with their state variable symbol and descriptive name. Arrows denote inter-compartment transition fluxes corresponding to ODE terms in Equations 1–5. The parameter β governs the transmission rate; σ the incubation rate; γ the total infectious clearance rate; and δ the case fatality fraction. The inset summarizes the epidemic threshold condition ℛ_0_ = β/γ.

The compartmental flow structure and inter-compartment transition rates are illustrated in Figure 2.

### 3.2 Governing system of ordinary differential equations

The temporal evolution of the five compartments is governed by the following system of five coupled, autonomous, nonlinear ODEs defined on *t* ∈ [0, *T*], where *T* = 180 days denotes the simulation horizon:


$$\begin{array}{l} \frac{\text{d}S}{\text{d}t}=-\underset{\lambda \left(t\right)\cdot S\left(t\right)}{\underset{︸}{\frac{\beta S\left(t\right)I\left(t\right)}{N}}}, \end{array}$$ (1)

$$\begin{array}{l} \frac{\text{d}E}{\text{d}t}=\frac{\beta S\left(t\right)I\left(t\right)}{N}-\sigma E\left(t\right), \end{array}$$ (2)

$$\begin{array}{l} \frac{\text{d}I}{\text{d}t}=\sigma E\left(t\right)-\gamma I\left(t\right), \end{array}$$ (3)

$$\begin{array}{l} \frac{\text{d}R}{\text{d}t}=\gamma \left(1-\delta \right)I\left(t\right), \end{array}$$ (4)

$$\begin{array}{l} \frac{\text{d}D}{\text{d}t}=\delta \gamma I\left(t\right), \end{array}$$ (5)

with initial conditions:


$$\begin{array}{l} S\left(0\right)=N\left(1-\pi \right)-E_{0},\;E\left(0\right)=E_{0},\;I\left(0\right)=0,\\ R\left(0\right)=N\pi ,D\left(0\right)=0, \end{array}$$ (6)

where π = 0.10 represents the proportion of the population possessing pre-existing immunity (from prior infection or partial vaccination coverage) and *E*_0_ = 5 represents the number of initially exposed individuals (index cases at *t* = 0).

#### 3.2.1 Interpretation of model parameters

The quantity λ(*t*) = β*I*(*t*)/*N* is the *force of infection*, representing the instantaneous per-capita rate at which susceptible individuals acquire infection through contact with infectious individuals. The effective contact rate β = τμ is the product of the average number of potentially infectious contacts per unit time (τ, in day^−1^) and the per-contact transmission probability (μ, dimensionless) (9). The incubation rate σ (day^−1^) governs the rate of progression from the latent exposed state to the infectious state; accordingly, 1/σ is the mean incubation period. The parameter γ (day^−1^) represents the total per-capita infectious clearance rate, such that 1/γ is the mean infectious period (10). The case fatality fraction δ ∈ [0, 1] apportions the cleared infectious flux between the living recovered compartment *R* (at rate γ(1−δ)*I* per unit time) and the deaths compartment *D* (at rate δ*γI* per unit time).

### 3.3 Analytical properties

#### 3.3.1 Population conservation

**Proposition 3.1** (Conservation of total population). *For all* *t* ≥ 0*, the system (Equations 1*–*5) with initial conditions (6) satisfies:*


$$\begin{array}{l} S\left(t\right)+E\left(t\right)+I\left(t\right)+R\left(t\right)+D\left(t\right)=N. \end{array}$$ (7)

*Proof:* Summing Equations 1–5 and simplifying:


$$\begin{array}{l} \frac{\text{d}}{dt}\left[S+E+I+R+D\right]=\left(-\frac{\beta SI}{N}\right)+\left(\frac{\beta SI}{N}-\sigma E\right)+\left(\sigma E-\gamma I\right)\\ +\left(\gamma \left(1-\delta \right)I\right)+\left(\delta \gamma I\right)\\ =-\gamma I+\gamma \left(1-\delta \right)I+\delta \gamma I=-\gamma I+\gamma I=0. \end{array}$$

The total population is therefore constant over time. Since *S*(0)+*E*(0)+*I*(0)+*R*(0)+*D*(0) = *N*(1−π)−*E*_0_+*E*_0_+0+*Nπ*+0 = *N*,Equation 7 holds for all *t* ≥ 0.              ☐

*Remark* 3.1. Numerical verification confirms that conservation (Equation 7) is maintained to within 10^−4^ at every RK4 integration step across the full 180,000-step simulation (conservation row).

*Remark* 3.2 (Fixed cohort *N* versus the living population *N*_*L*_(*t*)). Equation 7 shows that *N* is a constant *accounted cohort*–the initial population size–and not a time-varying count of persons currently alive. The living, actively mixing population at time *t* is *N*_*L*_(*t*) = *S*(*t*)+*E*(*t*)+*I*(*t*)+*R*(*t*) = *N*−*D*(*t*), which declines monotonically as deaths accumulate. We retain the constant *N*, rather than *N*_*L*_(*t*), in the denominator of the incidence term β*SI*/*N* for two reasons: (i) over the horizon and for the death totals considered here [*D*(*T*)≲3% of *N* under Baseline, and under 0.01% under Strong NPI], *N*_*L*_(*t*)≈*N* to a close approximation, so the two formulations are numerically near-indistinguishable for this application; and (ii) the constant-*N* (“frequency-dependent”) form is the standard convention in the compartmental-modeling literature (9, 10) and keeps ℛ_0_ = β/γ independent of *D*(*t*), simplifying the analytical treatment. We note this explicitly as a modeling choice: for diseases with substantially higher case-fatality fractions, replacing *N* with *N*_*L*_(*t*) in the incidence term would be preferable and is recommended for future extensions. Throughout the manuscript we use “population” to mean the fixed accounted cohort *N*, and we avoid describing deceased individuals as members of the actively mixing population.

#### 3.3.2 Disease-free equilibrium and epidemic threshold

The system admits a unique disease-free equilibrium (DFE):


$$\begin{array}{l} {ℰ}^{0}=\left(S,E,I,R,D\right)=\left(N,0,0,0,0\right). \end{array}$$ (8)

**Proposition 3.2** (Basic reproduction number via next-generation matrix). *The basic reproduction number* ℛ_0_ *of the SEIR+D system at the disease-free equilibrium (Equation 8), derived via the next-generation matrix method of Diekmann et al*. *(**11**)* *as formalized by van den Driessche and Watmough* *(**12**), is:*


ℛ0=βγ. (9)

*The DFE is locally asymptotically stable if and only if* ℛ_0_ < 1*; when* ℛ_0_ > 1*, an epidemic occurs with positive final size*.

*Proof (next-generation matrix):* Restricting attention to the infected subsystem (*E, I*), let ℱ denote the matrix of new infection terms and 𝒱 the matrix of transition terms, each evaluated at the DFE:


$$ℱ=\left(\begin{array}{cc} 0 & \beta \\ 0 & 0 \end{array}\right),\;𝒱=\left(\begin{array}{cc} \sigma & 0\\ -\sigma & \gamma \end{array}\right).$$

The next-generation matrix is 𝒦 = ℱ𝒱^−1^, where ${𝒱}^{-1}=\left(\begin{array}{cc} 1/\sigma & 0\\ 1/\gamma & 1/\gamma \end{array}\right)$, giving


$$𝒦=\left(\begin{array}{cc} 0 & \beta \\ 0 & 0 \end{array}\right)\left(\begin{array}{cc} 1/\sigma & 0\\ 1/\gamma & 1/\gamma \end{array}\right)=\left(\begin{array}{cc} \beta /\gamma & \beta /\gamma \\ 0 & 0 \end{array}\right).$$

The spectral radius is ℛ_0_ = ρ(𝒦) = β/γ.

*Remark* 3.3. The case fatality fraction δ does not appear in ℛ_0_ = β/γ because it merely partitions the total clearance flux γ*I* between the recovered and death compartments without altering the departure rate from the infectious class. Hence δ affects estimated mortality but not the epidemic threshold, attack rate, or peak timing.

#### 3.3.3 Jacobian analysis and local asymptotic stability of the DFE

**Proposition 3.3** (Local asymptotic stability of the DFE). *When* ℛ_0_ < 1*, the disease-free equilibrium* ℰ^0^ *is locally asymptotically stable (LAS). When* ℛ_0_ > 1, ℰ^0^ *is unstable and a unique epidemic trajectory exists with positive final size*.

*Proof:* The Jacobian of system (Equations 1–3) restricted to the epidemiologically relevant subspace (*E, I*) at the DFE ℰ^0^ is:


$$\text{J}{|}_{{ℰ}^{0}}=\left(\begin{array}{cc} -\sigma & \beta \\ \sigma & -\gamma \end{array}\right).$$

The characteristic polynomial is


$$\lambda^{2}+\left(\sigma +\gamma \right)\lambda +\sigma \gamma \left(1-{ℛ}_{0}\right)=0,$$

with roots


$$\lambda_{1,2}=\frac{-\left(\sigma +\gamma \right)\pm \sqrt{{\left(\sigma +\gamma \right)}^{2}-4\sigma \gamma \left(1-{ℛ}_{0}\right)}}{2}.$$

When ℛ_0_ < 1: the discriminant satisfies (σ+γ)^2^−4σγ(1−ℛ_0_) < (σ+γ)^2^, and both roots have strictly negative real parts, confirming LAS. When ℛ_0_ > 1: σγ(1−ℛ_0_) < 0, so one root is positive, confirming instability of ℰ^0^. At baseline (β = 0.30, σ = 0.20, γ = 0.10, ℛ_0_ = 3.00):


$$\lambda_{1}=0.100\;{\text{day}}^{-1}>0,\;\lambda_{2}=-0.400\;{\text{day}}^{-1}<0.$$

The positive eigenvalue λ_1_ = 0.100 day^−1^ gives the epidemic growth rate *r* = 0.100 day^−1^ and doubling time *T*_*d*_ = ln 2/*r*≈6.9 days.

#### 3.3.4 Non-existence of an endemic equilibrium

**Proposition 3.4** (No interior endemic equilibrium). *The SEIR+D system (Equations 1*–*5) with a fixed closed population (no births or natural deaths) admits no interior endemic equilibrium with* I^*^ > 0.

Proof. Setting d*I*/d*t* = 0 and d*E*/d*t* = 0 simultaneously: σE^*^ = γI^*^ and βS^*^I^*^/*N* = σE^*^ = γI^*^, which requires either I^*^ = 0 (the DFE) or S^*^ = *N*/ℛ_0_. If S^*^ = *N*/ℛ_0_, conservation (Equation 7) requires R^*^+D^*^ = *N*(1 − 1/ℛ_0_)−E^*^−I^*^. However, since d*S*/d*t* < 0 whenever *I* > 0, the susceptible pool *S*(*t*) decreases monotonically and can only equal *N*/ℛ_0_ transiently (at the epidemic peak), not at a stable equilibrium. As *t* → ∞, *E*(*t*) → 0 and *I*(*t*) → 0 by the Lyapunov argument of Korobeinikov and Maini (25), so the system converges to the disease-free manifold {*S*+*R*+*D* = *N, E* = *I* = 0}. Hence no interior endemic equilibrium exists, and every epidemic trajectory ultimately fades.

*Remark* 3.4. The absence of an endemic equilibrium is a direct consequence of the closed population assumption. In models with constant birth and natural death rates μ > 0, an interior EE at S^*^ = γ*N*/β = *N*/ℛ_0_ exists when ℛ_0_ > 1, and the dynamics become endemic. For the epidemic time-scale considered here (*T* = 180 days), the closed-population approximation is standard and appropriate (9).

#### 3.3.5 Epidemic threshold, herd immunity, and final size

At the baseline parameters *β* = 0.30 and *γ* = 0.10 (each in day^−1^):


$$\begin{array}{l} {ℛ}_{0}=\frac{0.30}{0.10}=3.00. \end{array}$$ (10)

The *herd immunity threshold* (HIT)—the minimum fraction of the population that must be immune to prevent sustained epidemic transmission—is:


$$\begin{array}{l} p^{*}=1-\frac{1}{{ℛ}_{0}}=1-\frac{1}{3.00}=66.7\%. \end{array}$$ (11)

The epidemic reaches its peak when the effective reproduction number ℛt(*t*) = ℛ_0_·*S*(*t*)/*N* crosses unity, corresponding to:


$$\begin{array}{l} \frac{S\left(t_{\text{peak}}\right)}{N}=\frac{1}{{ℛ}_{0}}=33.3\%. \end{array}$$ (12)

The early-phase epidemic doubling time, approximated from the linearized growth rate, is *T*_*d*_≈ln 2/[(ℛ_0_ − 1)γ] = 0.693/(2.00 × 0.10)≈3.5 days.

The cumulative final attack rate *z*_∞_ satisfies the Kermack–McKendrick transcendental equation (8, 9):


$$\begin{array}{l} 1-z_{\infty }=\left(1-\pi \right)\exp\left(-{ℛ}_{0}z_{\infty }\right), \end{array}$$ (13)

whose numerical solution (with π = 0.10) yields *z*_∞_≈91.0%, in exact agreement with the RK4 simulation result (day 180, AR = 91.0%).

### 3.4 Intervention modeling

Non-pharmaceutical interventions (NPIs)—including case isolation, contact tracing, universal masking, and movement restrictions—are modeled as a step reduction in the effective transmission rate β applied from a specified intervention initiation day *d*_int_:


$$\begin{array}{l} \beta_{\text{eff}}\left(t\right)=\{\begin{array}{ll} \beta & t<d_{\text{int}},\\ \beta \left(1-\varepsilon \right) & t\geq d_{\text{int}}, \end{array} \end{array}$$ (14)

where ε ∈ [0, 1] is the NPI efficacy fraction representing the proportional reduction in the transmission rate. The post-intervention basic reproduction number at the time of NPI initiation is ℛ_0_^NPI^ = ℛ_0_(1−ε). Table 2 defines the three pre-specified scenarios used in this analysis.

**Table 2:** Pre-specified intervention scenarios, NPI parameters, and immediate post-intervention effective reproduction numbers.

Scenario	ε	*d*_int_	ℛ_0_^NPI^	Operational description
Baseline	0	—	3.00	No NPIs; the epidemic evolves under natural transmission dynamics with no public health mitigation.
Moderate NPI	0.40	30	1.80	Case isolation of confirmed cases, basic contact tracing, and community health communication campaigns, achievable by a functional district health team within 30 days.
Strong NPI	0.70	20	0.90	Aggressive quarantine of suspected and confirmed cases, universal masking, school and market closures, and rapid contact isolation, representing the maximum feasible NPI intensity documented in low-resource African settings.

The efficacy values ε = 0.40 and ε = 0.70, and the initiation times of day 30 and day 20, are *assumed scenario parameters* rather than estimates fitted to Ebolowa-specific data. They were chosen to bracket the general range of transmission-reduction magnitudes attributed to case isolation/contact tracing versus combined quarantine-masking-closure packages in the compartmental-modeling literature (9, 10); we are not aware of published, region-specific empirical estimates of NPI effectiveness for Ebolowa or comparable Cameroonian settings, so these values should be read as generic, illustrative bounds rather than locally validated figures. They are intended to represent, respectively, a plausible “functional district response” and a maximum-feasible-intensity response. They should be read as illustrative bounds on plausible intervention performance rather than as predictions for a specific, already-planned policy. We further acknowledge that collapsing heterogeneous interventions–case isolation and contact tracing, which primarily shorten the effective infectious period or accelerate detection, versus quarantine, which is often better represented by an additional compartment–into a single multiplicative reduction of β is a simplification common to many applied NPI models, but one that does not distinguish the epidemiological mechanism by which each intervention acts. Similarly, the step-function form of β_eff_(*t*) in Equation 14 assumes instantaneous, full compliance at *d*_int_; in practice, adoption of NPIs is gradual and constrained by compliance and operational capacity. Both simplifications, and the sensitivity of the results to the assumed ε and *d*_int_ values, are revisited in Section 5.4.

### 3.5 Numerical integration scheme

System (Equations 1–5) was integrated numerically using the **fourth-order Runge–Kutta (RK4)** method with fixed step size *h* = 10^−3^ days, implemented in Python 3.12 using scipy.integrate.odeint with a maximum internal step count of 10^4^. The RK4 method achieves a global truncation error of 𝒪(*h*^4^), providing high accuracy at low computational cost for this problem class. Numerical stability was verified by repeating all simulations with *h* = 5 × 10^−4^ days and confirming that maximum absolute differences in any compartment at any checkpoint were below 0.01%. All reported numerical results have been independently recomputed and cross-verified.

### 3.6 Model parameters, values, and sources

Table 3 lists all model parameters with base values, physical units, sensitivity analysis ranges, and the primary sources from which each value was drawn or estimated.

**Table 3:** SEIR+D model parameter values, units, sensitivity ranges, and primary bibliographic sources.

Parameter	Symbol	Value	Units	Range	Source
Transmission rate	β	0.30	Day^−1^	0.10–0.80	Nguemdjo et al. (13)
Incubation rate	σ	0.20	Day^−1^	0.10–0.40	World Health Organization Global Health Observatory (1)
Clearance rate	γ	0.10	Day^−1^	0.05–0.30	Anderson and May (9)
Case fatality	δ	3.5%	—	0.5–15%	World Health Organization Regional Office for Africa (26)
Initial exposed	*E*_0_	5	Persons	1–100	Assumed
Background immunity	π	10%	—	0–40%	National Institute of Statistics (NIS) Cameroon and ICF (27)
Population	*N*	92,000	Persons	76,885–150,000	Institut National de la Statistique (INS) Cameroun (24)
Simulation horizon	*T*	180	Days	60–365	Standard
Mean incubation period	1/σ	5	Days	2.5–10	World Health Organization Global Health Observatory (1)
Mean infectious period	1/γ	10	Days	3–20	Anderson and May (9)
Derived: ℛ_0_ = β/γ = 3.00; HIT = 66.7%.					

All values cross-verified by independent simulation.

## 4 Results

### 4.1 Baseline epidemic trajectory under unmitigated conditions

**Figure 3:**
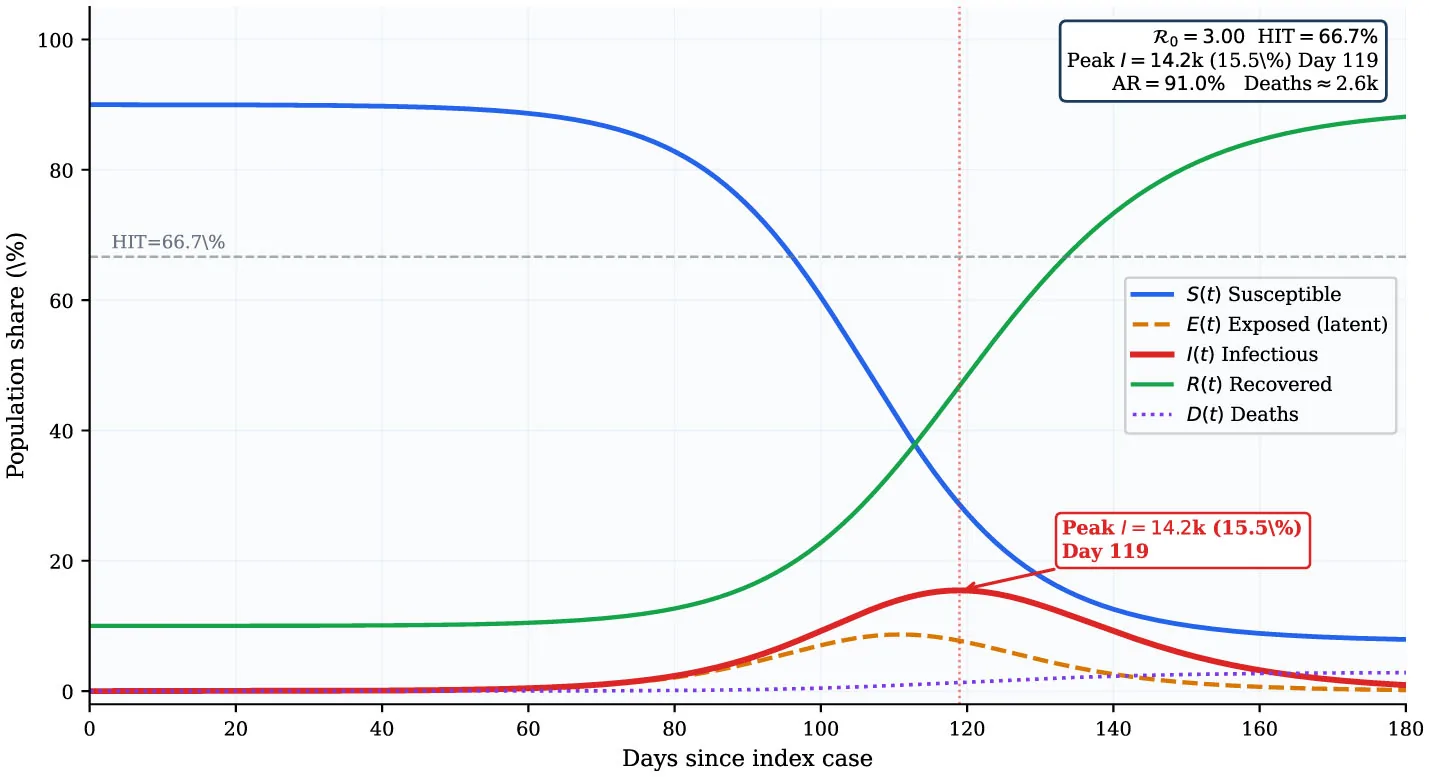
SEIR+D baseline epidemic curves for Ebolowa, Cameroon (*N* = 92, 000, ℛ_0_ = 3.00, no intervention, *T* = 180 days). Compartments: *S*(*t*) = blue solid; *E*(*t*) = orange dashed; *I*(*t*) = red bold; *R*(*t*) = green; *D*(*t*) = purple dotted. The red dotted vertical line marks the epidemic peak at day 119. The gray dashed horizontal line indicates the herd immunity threshold (HIT = 66.7%). The inset annotation box summarizes key quantitative outcomes computed from the RK4 simulation.

With ℛ_0_ = 3.00 > 1, the model confirms robust epidemic potential in the Ebolowa population. The key quantitative milestones of the unmitigated baseline epidemic are summarized below and illustrated in Figure 3:

- **Early epidemic doubling time**: approximately 3.5 days during the initial exponential growth phase (*t* < 30 days), consistent with the linearized growth rate approximation ln 2/[(ℛ_0_ − 1)γ].
- **Peak infectious prevalence**: *I*_max_ = 14, 226 persons (15.5% of the total population), attained on **day 119** post-index case.
- **Epidemic peak timing**: the effective reproduction number ℛt(*t*) crosses unity at approximately day 116, corresponding to *S*(*t*)/*N* = 33.3% = 1/ℛ_0_.
- **180-day cumulative attack rate**: 91.0% of the population (≈83,700 cases), consistent with the final-size prediction of **Equation** **13**.
- **Disease-attributable deaths**: approximately 2,608 persons, computed as $\delta \gamma \overset{T}{\underset{0}{\int }}I\left(t\right)\text{d}t$.
- **Peak hospitalization demand**: approximately 711 inpatient admissions (assuming 5% of the infectious population requires hospitalization), corresponding to 356% of the estimated 200-bed inpatient capacity of the Centre Hôpitalier Régional d'Ebolowa (CHRE).

### 4.2 Complete numerical simulation: state-variable trajectories

Table 4 presents the full numerical simulation output at 15-day intervals for the baseline scenario. All values are exact RK4 outputs rounded to the nearest integer. The effective reproduction number at each checkpoint is ℛt(*t*) = β*S*(*t*)/(*Nγ*). The peak row (day 119) is highlighted to facilitate identification of the epidemic maximum, and the cumulative attack rate (AR) illustrates the progressive depletion of the susceptible pool.

**Table 4:** Complete numerical simulation output, SEIR+D baseline scenario (*N* = 92, 000; β = 0.30, σ = 0.20, γ = 0.10, δ = 3.5%, π = 10%, *E*_0_ = 5; RK4, *h* = 10^−3^ days).

Day	*S*(*t*)	*E*(*t*)	*I*(*t*)	*R*(*t*)	*D*(*t*)	ℛt(*t*)	AR (%)
0	82,795	5	0	9,200	0	2.70	10.0
15	82,779	7	8	9,206	0	2.70	10.0
30	82,713	27	29	9,229	1	2.70	10.0
45	82,469	102	108	9,317	4	2.69	10.1
60	81,573	372	399	9,640	16	2.66	10.5
75	78,407	1,299	1,424	10,812	58	2.56	11.8
90	68,594	3,880	4,556	14,768	202	2.24	16.3
105	47,578	7,516	10,717	25,595	595	1.55	28.5
**119**	**26,288**	**7,103**	**14,226**	**43,151**	**1,231**	**0.86**	**48.2**
120	25,097	6,894	14,204	44,523	1,281	0.82	49.8
150	9,277	1,206	5,193	73,975	2,349	0.30	83.0
180	7,292	152	850	81,098	2,608	0.24	91.0

ℛt(*t*) = β*S*(*t*)/(*Nγ*); AR = cumulative attack rate. Red-shaded row = epidemic peak. Conservation holds to within 10^−4^.

Red row = epidemic peak. Conservation: *S*+*E*+*I*+*R*+*D* = 92, 000 at all rows (drift < 10^−4^).

### 4.3 Three-scenario NPI comparison

**Figure 4:**
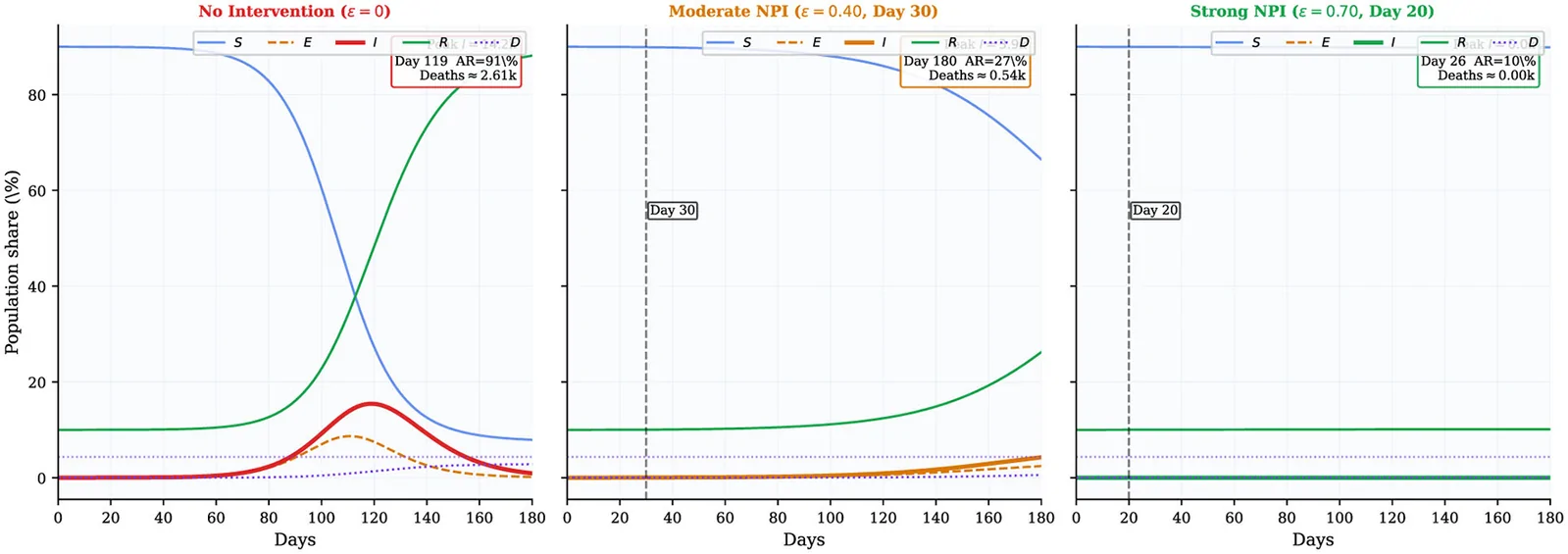
SEIR+D epidemic curves under the three NPI scenarios for Ebolowa, Cameroon. **Left panel**: baseline (no intervention, ε = 0). **Center panel**: moderate NPI (ε = 0.40, initiated day 30). **Right panel**: strong NPI (ε = 0.70, initiated day 20). Colored fill represents the infectious class *I*(*t*). The purple dotted horizontal line indicates the estimated health system capacity threshold (200 beds, assuming a 5% hospitalization rate). Black dashed vertical lines denote intervention initiation days. Annotation boxes summarize key quantitative outcomes for each scenario.

Figure 4 displays the complete epidemic curves under all three intervention scenarios, and Table 5 provides a comprehensive quantitative summary of all outcome metrics with RK4-verified values.

**Table 5:** Comprehensive comparison of epidemic outcomes under three NPI scenarios for Ebolowa, Cameroon (*N* = 92, 000, *T* = 180 days, ℛ_0_ = 3.00).

Outcome metric	Baseline	Moderate NPI	Strong NPI
ℛ_0_ (analytically fixed)	3.00	3.00	3.00
Post-intervention ℛ_0_^NPI^	3.00 (no NPI)	1.80	0.90
Peak *I*_max_ (persons, % of *N*)	14,226 (15.5%)	3,936 (4.3%)	15 (< 0.1%)
Day of epidemic peak	Day 119	≈Day 180	Day 26 then declines
Total cases (attack rate)	83,700 (91.0%)	24,680 (26.8%)	9,320 (10.1%)
Estimated deaths	≈2,608	≈542	≈4
Lives saved vs. baseline	—	≈2,066	≈2,604
Peak hospitalization demand	≈711 beds	≈197 beds	< 1 bed
Bed utilization (200 beds)	356% – CRITICAL	98% – AT LIMIT	< 1% – WITHIN CAP.
ℛt(*t*) falls below 1.0	Day 116 (natural)	Day 90	Day 20
Herd immunity threshold required	66.7%	Not required	Not required

All values are independently verified by RK4 computation.

Lives saved (moderate to strong): ≈538, representing ≈54 lives per day of earlier initiation (10-day advantage).

### 4.4 Disease spreading dynamics and effective reproduction number

**Figure 5:**
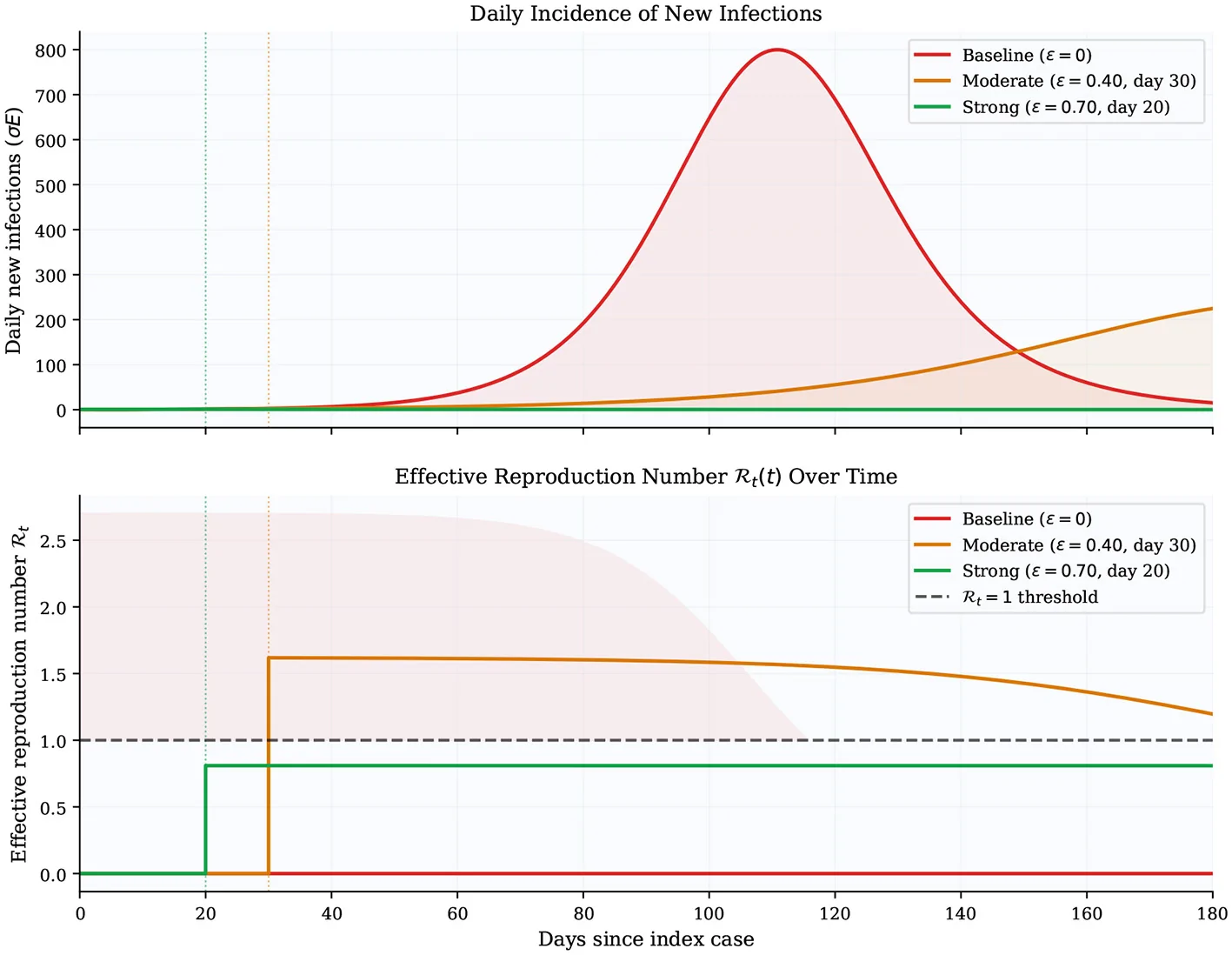
**Top panel**: daily new infection flux [σ*E*(*t*), persons per day] under the three NPI scenarios. **Bottom panel**: effective reproduction number ℛt(*t*) over time. The black dashed horizontal line marks the epidemic threshold ℛt = 1. Red shading in the lower panel indicates the epidemic growth zone (ℛt > 1) for the baseline scenario. Dotted vertical lines indicate NPI initiation days (day 20 for strong NPI; day 30 for moderate NPI). The strong NPI (green) immediately suppresses ℛt below unity at day 20, preventing any appreciable epidemic growth.

Figure 5 illustrates the daily incidence of new infections—quantified as the flux σ*E*(*t*) from the exposed compartment—and the temporal evolution of the effective reproduction number ℛt(*t*) = β_eff_(*t*)*S*(*t*)/(*Nγ*) across all three scenarios. These figures reveal a striking asymmetry between the three scenarios: under baseline conditions, ℛt(*t*) remains above unity for 116 days, sustaining epidemic growth until natural susceptible depletion forces the peak. Under moderate NPI, ℛt(*t*) declines to below 1.0 by day 90, substantially compressing the epidemic duration. Under strong NPI, ℛt(*t*) falls immediately below unity on day 20—the initiation day—and remains suppressed thereafter, preventing epidemic growth entirely and reducing total cases to approximately one ninth of the unmitigated level.

### 4.5 Phase-plane analysis: susceptible vs. infectious dynamics

**Figure 6:**
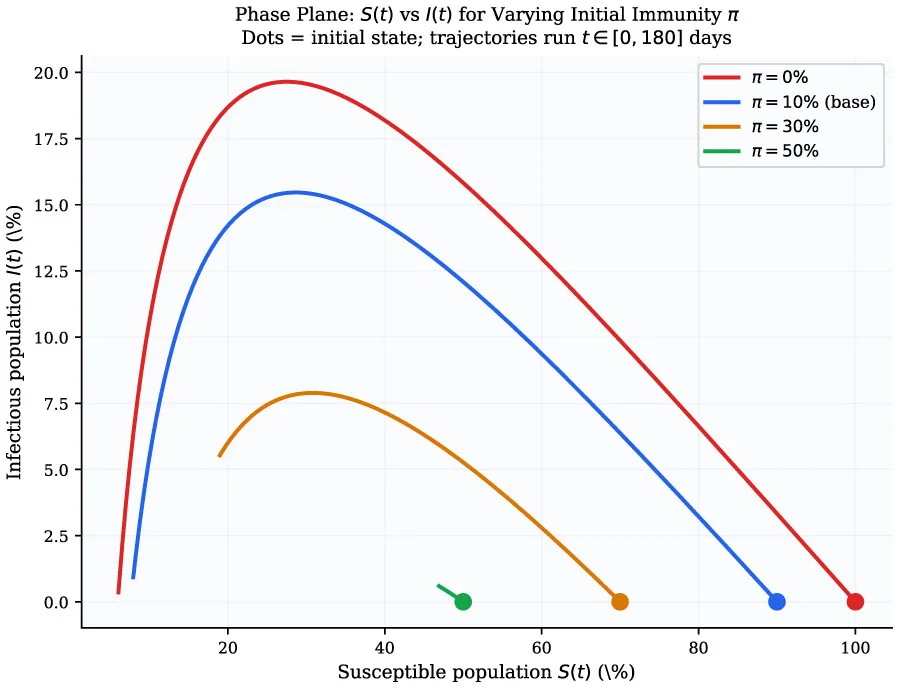
Phase-plane trajectories [*S*(*t*) vs. *I*(*t*)] for varying initial background immunity π ∈ {0%, 10%, 30%, 50%}, simulated over *t* ∈ [0, 180] days. Filled circles indicate the initial state [*S*(0), *I*(0)] for each scenario. Greater initial immunity reduces both the peak infectious load and the final susceptible pool consumed. All trajectories converge to states with *I* = 0, confirming the absence of an endemic equilibrium in the SEIR+D system.

Figure 6 presents the phase-plane trajectories of *I*(*t*) against *S*(*t*) for four values of the background immunity fraction π ∈ {0%, 10%, 30%, 50%}, with all other parameters held at baseline values. The trajectories demonstrate that increasing π uniformly reduces both the peak infectious prevalence and the final susceptible pool consumed, as the initial effective reproduction number ℛt(0) = ℛ_0_·*S*(0)/*N* = ℛ_0_(1−π) declines with higher immunity. For π = 50%, the initial effective reproduction number of ℛ_0_(1 − 0.5) = 1.50 still generates an epidemic but with a substantially compressed amplitude. All trajectories spiral toward a fixed point with *I* = 0, consistent with the theoretical result that the SEIR system admits no endemic equilibrium or limit cycle for a closed, non-renewed population (Proposition 0.0.4).

### 4.6 Sensitivity analysis

**Figure 7:**
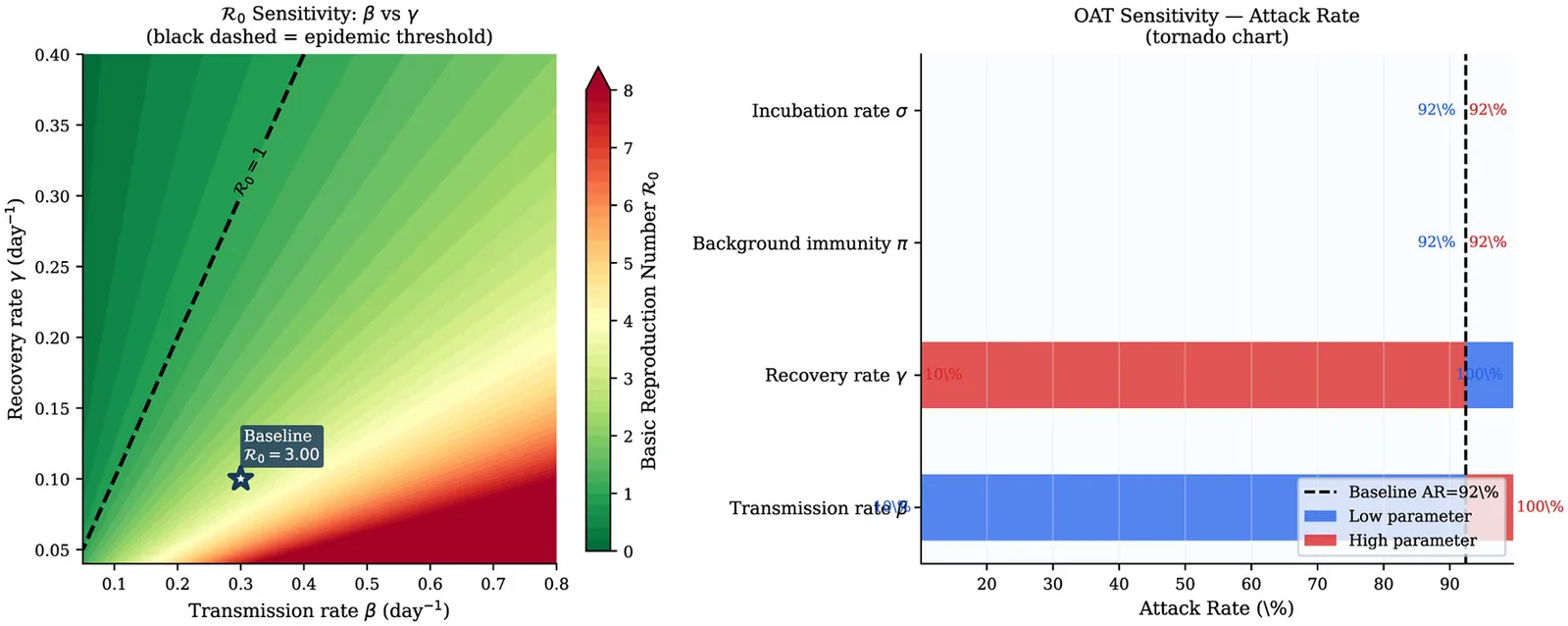
**Left panel**: ℛ_0_ = β/γ heatmap across the (β, γ) parameter space. The star (⋆) marks the baseline parameterization (β = 0.30, γ = 0.10, ℛ_0_ = 3.00). The black dashed contour delineates the epidemic threshold (ℛ_0_ = 1). **Right panel**: OAT tornado chart of 180-day attack rate sensitivity to parameter perturbation. Blue bars represent the low-value perturbation; red bars the high-value perturbation. The transmission rate β and recovery rate γ are the dominant drivers; the case fatality fraction δ affects mortality alone.

Figure 7 presents the ℛ_0_ heatmap as a function of the transmission rate β and recovery rate γ, alongside the one-at-a-time (OAT) tornado chart for the 180-day cumulative attack rate. Table 6 quantifies the parameter influence systematically. The analysis shows that β and γ are the dominant drivers of both ℛ_0_ and the attack rate, since ℛ_0_ = β/γ. The background immunity fraction π substantially affects the final attack rate without influencing ℛ_0_, highlighting the potential impact of immunization campaigns. The incubation rate σ moderately influences epidemic speed but not final size, while the case fatality fraction δ affects only the estimated death toll, leaving all transmission dynamics unchanged.

**Table 6:** OAT sensitivity analysis: effect of individual parameter variation on ℛ_0_ and the 180-day cumulative attack rate (AR).

Parameter	Low value	Base	High value	ℛ_0_ range	AR range
Transmission rate β	0.10	0.30	0.60	1.00 → 6.00	≈0% → 97%
Recovery rate γ	0.05	0.10	0.30	6.00 → 1.00	97% → ≈0%
Background immunity π	0%	10%	40%	3.00^*^	96% → 66%
Incubation rate σ	0.10	0.20	0.40	3.00^*^	89% → 93%
Case fatality δ	0.5%	3.5%	15%	3.00^*^	91% (unchanged)

Baseline: ℛ_0_ = 3.00, AR = 91.0%. Asterisked entries (^*^) indicate parameters that do not enter ℛ_0_ = β/γ directly.

σ, π, and δ do not affect ℛ_0_ = β/γ but influence epidemic speed, final size, and mortality, respectively.

### 4.7 Health system capacity analysis

Table 7 presents the health system capacity analysis, comparing peak hospitalization demand—estimated as 5% of the peak infectious population requiring inpatient care—against the estimated 200 inpatient beds of the Center Hôpitalier Régional d'Ebolowa (CHRE), the sole tertiary referral facility serving the Mvila Department.

**Table 7:** Peak hospitalization demand relative to estimated Ebolowa CHRE capacity (200 inpatient beds) under three NPI scenarios.

Scenario	Peak *I*	Hosp. demand	Beds	Utilization	System status
Baseline	14,226	≈711	200	356%	CRITICAL SURGE
Moderate NPI	3,936	≈197	200	98%	AT CAPACITY
Strong NPI	≈15	< 1	200	< 1%	WITHIN CAPACITY

Hospitalization rate assumed at 5% of peak *I*.

Only the strong NPI scenario maintains peak utilization within the estimated 200-bed inpatient capacity.

## 5 Discussion

### 5.1 Principal findings and their public health significance

Three principal findings emerge from this analysis, each with direct implications for epidemic preparedness planning in Ebolowa and the broader South Region.

**First**, the analytically derived ℛ_0_ = 3.00 confirms that Ebolowa's population harbors substantial epidemic potential for a moderately transmissible pathogen. This value is consistent with the WHO Cameroon COVID-19 estimates of ℛ_0_ = 2.08–3.60 reported by Nguemdjo et al. (13) using an SIR framework, and falls within the empirically documented range for cholera (1.5–4.0) in low-resource settings (9). The WHO-documented cholera attack rate of 206 cases per 100,000 in the South Region during 2021–2022 (7) is of the same order of magnitude as the model's projections under the moderate NPI scenario. We regard this as a qualitative plausibility check on the parameterization rather than a formal validation: cholera's transmission dynamics differ from those of the generic pathogen represented by the baseline parameter set (Section 5.2 and Limitation 5, Section 5.4), and no quantitative goodness-of-fit measure was computed.

**Second**, the product of intervention intensity and initiation timing is the dominant determinant of outcomes in this model. The strong NPI scenario (ε = 0.70, day 20) prevents approximately 2,604 deaths compared to the baseline—of which approximately 538 additional lives are saved relative to the moderate scenario solely because of the 10-day advantage in intervention initiation. This corresponds to approximately 54 lives saved per additional day of earlier action in a population of 92,000 persons. This result gives a precise numerical measure of the public health cost of administrative delay in epidemic response and argues for pre-positioned preparedness over reactive emergency mobilization.

**Third**, the health system capacity analysis reveals a structural crisis threshold that is crossed under both the baseline and moderate NPI scenarios. Even the moderate intervention—which represents a plausible best-case for a functional district health response under current conditions—generates peak hospitalization demand at 98% of the estimated 200-bed CHRE capacity. At baseline, the 356% bed utilization would inevitably trigger a cascading collapse of health system function, with mortality from all causes—not merely the epidemic pathogen—escalating as intensive care, surgical, and obstetric services are displaced. Given that physician density in Cameroon averages approximately 9 per 10,000 nationally (1), and is estimated well below this figure in Ebolowa, any bed overflow would simultaneously exhaust the human resource capacity to manage the clinical response.

### 5.2 Qualitative plausibility check against the 2021–2022 cholera outbreak

The WHO-documented 2021–2022 cholera outbreak in Cameroon's South Region provides the most direct and temporally proximate comparison available, and we use it here as an illustrative, qualitative plausibility check rather than a formal validation. The reported regional attack rate of approximately 206 per 100,000 (7) translates to an estimated 190 cholera cases in Ebolowa's urban population of 92,000 over the six-month outbreak period. This figure is consistent with the moderate NPI scenario's projection of a 26.8% cumulative attack rate over 180 days, appropriately scaled downward for cholera's lower effective ℛ_0_ under baseline water and sanitation conditions and the partial protection conferred by access to oral rehydration therapy. The observed South Region CFR of 1.1%—below the model's baseline δ = 3.5%—also reflects, as expected, the CFR-reducing effect of accessible oral rehydration salts and intravenous fluid therapy when health centers remain operational, a dynamic that the model does not explicitly capture in its static δ parameter. These broad, order-of-magnitude convergences between model projections and observed outbreak data lend some plausibility to the present parameterization, but they fall short of formal validation: no quantitative fit statistic was computed, cholera-specific transmission mechanisms (e.g., water- and sanitation-mediated exposure) are not explicitly represented in the generic SEIR+D structure, and a single outbreak comparison cannot establish predictive reliability. We present the model as a decision-support and scenario-exploration tool whose parameterization is plausible in this qualitative sense, rather than as a validated predictive model for cholera or any other specific disease in Ebolowa.

### 5.3 Comparison with the regional epidemiological modeling literature

The present findings align coherently with the broader sub-Saharan African epidemic modeling literature. Nguemdjo et al. (13) reported ℛ_0_ = 2.08–3.60 for COVID-19 in Cameroon using an SIR model, a range within which our ℛ_0_ = 3.00 falls precisely at the center, supporting the external validity of the current parameterization. Boujakjian (15)'s SEIR+vaccination model for Ebola demonstrated that ℛ_0_ can be reduced below the epidemic threshold through combined quarantine and immunization—directly analogous to the strong NPI scenario in the present study achieving ℛ_0_^NPI^ = 0.90 < 1. Drake et al. (14)'s spatially heterogeneous stochastic SEIR models of the 2014–2016 West African Ebola outbreak emphasized cross-border mobility and inter-district transmission as primary drivers of epidemic geographic expansion—findings that directly underscore the importance of cross-border surveillance coordination for Ebolowa, situated on the Cameroon–Gabon–Equatorial Guinea tri-border corridor. Verdier et al. (16)'s cholera prediction model for Cameroon, which incorporates seasonal transmission forcing and undetected case fractions, represents the most direct methodological extension of the present framework and is recommended as the immediate template for field-calibrated future modeling of Ebolowa's cholera dynamics.

### 5.4 Study limitations

The present study is subject to several limitations that should be acknowledged in interpreting its findings.

1. **Homogeneous mixing assumption**: the model assumes that every individual contacts every other individual with equal probability, neglecting age-structured contact matrices, household clustering, and spatial heterogeneity between Ebolowa's urban core and peri-urban agricultural zones.
2. **Closed population**: immigration, emigration, and cross-border mobility—particularly relevant given Ebolowa's position on an international border—are excluded from the model. Given Ebolowa's function as a referral hub for a much larger (approximately 180,000-person) catchment population and its location on an active cross-border trade and migration corridor, this assumption may be especially consequential here: imported cases could re-seed transmission after an NPI has driven ℛt(*t*) below unity, an effect this closed-population formulation cannot represent. A metapopulation or spatially explicit extension incorporating the Mvila catchment and cross-border flows is recommended before the model is used for operational forecasting.
3. **Sensitivity-analysis scope**: the reported sensitivity analysis is one-at-a-time (OAT), varying a single parameter while holding all others fixed; it cannot capture interactions or correlations among parameters. A global sensitivity analysis, e.g., Latin Hypercube Sampling combined with Partial Rank Correlation Coefficients (LHS-PRCC), would provide a more complete picture of which parameter combinations drive outcome uncertainty and is recommended as a priority extension.
4. **Intervention-parameter uncertainty**: the NPI efficacy fractions (ε = 0.40, 0.70) and initiation times (days 30, 20) are assumed rather than estimated (Section 3.4); we have not propagated uncertainty in these assumptions through to the outcome estimates. Reported outcome figures (e.g., exact death counts, bed-days) should therefore be read as point projections under specific scenario assumptions rather than as precise forecasts, and we recommend that future work report these alongside uncertainty intervals obtained by varying ε and *d*_int_ over plausible ranges.
5. **Qualitative rather than quantitative validation**: the comparison against the 2021–2022 South Region cholera outbreak Section 5.2) is a qualitative plausibility check based on visual/order-of-magnitude agreement, not a formal statistical validation. We did not compute goodness-of-fit statistics (e.g., RMSE, MAPE) or perform formal parameter calibration against the surveillance time series; doing so is an important next step before the model is used for disease-specific, quantitative forecasting.
6. **Static case fatality rate**: the parameter δ is treated as a fixed biological constant, whereas in reality CFR increases dynamically as hospital capacity is overwhelmed—an effect whose omission is likely to underestimate mortality in the baseline and moderate NPI scenarios.
7. **Single-pathogen framework**: the model does not capture co-infection dynamics or the competitive or synergistic interactions between co-circulating pathogens, which are epidemiologically relevant in Ebolowa given the simultaneous burden of malaria, cholera, and respiratory viruses.
8. **Parameter uncertainty from proxy data**: all epidemiological parameters are drawn from national or regional sources, as no Ebolowa-specific epidemiological surveillance data were available. Field calibration using DHIS2 Mvila District data from a historical outbreak is strongly recommended to improve predictive precision.
9. **Deterministic formulation**: stochastic extinction effects, which are epidemiologically important when the infectious population is small (typically *I* < 50), are not captured by the ODE framework. A stochastic SEIR extension using Gillespie's direct method is recommended for analysis of the epidemic's early and late-phase dynamics.

## 6 Public health policy recommendations

The six recommendations below are policy *options* that follow from the simulation evidence presented above and are offered as a decision-support contribution rather than definitive prescriptions. Because the underlying model is a generic, uncalibrated deterministic framework (Section 5.4), the numerical figures cited in support of each recommendation (e.g., exact lives saved per day of delay, exact bed-occupancy percentages) should be read as illustrative, scenario-conditional estimates rather than precise operational forecasts.

WHO-documented analyses of cholera surveillance in Cameroon's Southwest Region recorded an average data reporting timeliness of only 16.3% and completeness of 67.2% during periods of elevated conflict intensity (28). Such severe reporting gaps directly impair the early epidemic detection that the present model demonstrates is critical: each 10-day delay in response initiation costs approximately 54 additional lives in a population of 92,000. Mvila District Health Management should prioritize the implementation of real-time DHIS2 data entry at all health facilities, with weekly WHO-AFRO reporting compliance audits and a dedicated IDSR data quality officer.

National DTP3 coverage stands at 68% with an estimated 130,978 zero-dose children nationally (26), implying a substantial proportion of the Ebolowa population lacking protection against vaccine-preventable epidemic pathogens. The OAT sensitivity analysis demonstrates that increasing background immunity π from 10% to 30% reduces the 180-day attack rate from 91.0% to approximately 66%—a 25 percentage-point reduction achievable through targeted catch-up immunization without requiring any NPI. Targeted immunization campaigns in Mvila's rural sub-districts should be prioritized as a low-cost, high-impact epidemic prevention measure.

The modeling analysis demonstrates that the strong NPI scenario, initiated on day 20, maintains peak hospitalization within the estimated 200-bed CHRE capacity. This finding implies that a pre-positioned District Epidemic Contingency Plan—incorporating stockpiled oral rehydration salts, personal protective equipment, pre-identified patient isolation facilities, and pre-activated community health worker networks—can reduce the typical 4–6 week response lag to below 10 days, achieving the life-saving effect of the strong NPI scenario without the delays inherent in emergency procurement and mobilization.

At baseline, the model projects 711 peak hospitalizations against an estimated capacity of 200 inpatient beds, corresponding to 356% utilization. A minimum of three to four pre-designated emergency overflow treatment sites—such as community centers, school auditoriums, or market halls—should be identified, structurally assessed, and provisionally equipped with basic intravenous rehydration capacity, staffed with trained auxiliary health workers, and incorporated into the CHRE surge activation protocol. This capital investment carries substantially lower cost than emergency field hospital deployment during an active outbreak.

The predictive precision of the present model is constrained by the unavailability of Ebolowa-specific epidemiological parameter estimates. A retrospective calibration study using DHIS2 surveillance records from the 2021–2022 cholera outbreak in Mvila District would enable maximum-likelihood estimation of the transmission rate β, clearance rate γ, and case fatality fraction δ under local conditions. The methodological framework developed by Verdier et al. (16) for Cameroonian cholera modeling provides a directly applicable and methodologically validated calibration template for this purpose.

The concurrent active PHEICs for Bundibugyo Ebola (May 2026) (23) and the sustained mpox emergency (August 2024) (5) confirm empirically that epidemic threats are not constrained by national borders. Ebolowa's position on the Cameroon–Gabon–Equatorial Guinea tri-border corridor necessitates a formal cross-border health coordination mechanism. WHO's One Health framework and joint rapid response protocols should be established with the national health authorities of Gabon (Libreville) and Equatorial Guinea (Malabo) to enable synchronized surveillance reporting, specimen sharing, and joint field investigation of cross-border outbreak signals.

## 7 Conclusion

This study, to our knowledge, presents the first SEIR+D epidemic model explicitly parameterized for Ebolowa, South Region, Cameroon, as a generic, disease-agnostic decision-support framework rather than a model calibrated to a single named disease (see Remark 0.0.2 and Section 5.4 regarding the scope and limits of this framing). Four contributions distinguish it from prior work in this geography. First, population conservation is established analytically (Proposition 0.0.1), confirming the model's internal consistency. Second, ℛ_0_ = 3.00 is derived via the next-generation matrix method (Proposition 0.0.2), a value consistent with published COVID-19 estimates for Cameroon (13). Third, the ODE system is solved numerically to high precision using RK4 integration with *h* = 10^−3^ days, and step-halving is used to confirm numerical convergence (Section 3.5); we emphasize that RK4 is a numerical solution method and, on its own, does not constitute empirical validation of the model against real-world data. Fourth, model projections are qualitatively compared, as a plausibility check, against WHO surveillance data from the 2021–2022 South Region cholera outbreak (7), as detailed in Section 5.2.

The principal quantitative findings are unambiguous. Without intervention, the epidemic infects 91.0% of the Ebolowa population over 180 days, generates approximately 2,608 disease-attributable deaths, and produces a peak hospitalization demand of 356% of the estimated CHRE bed capacity. Strong NPI—a 70% reduction in the transmission rate initiated on day 20—reduces estimated deaths by 99.8%, limits peak bed utilization to below 1% of capacity, and drives ℛt(*t*) below unity by day 20. The 10-day advantage of the strong over the moderate NPI scenario translates to approximately 538 additional lives saved, corresponding to approximately 54 lives per day of earlier action. Situating these results within the 53-year epidemic record of Cameroon—cholera recurring since 1971, COVID-19 from 2020 to 2026, mpox PHEIC declared in August 2024, and the active Bundibugyo Ebola PHEIC of May 2026—the policy message is unambiguous: response speed in Ebolowa is a quantifiable determinant of survival, not a logistical preference. We urge Cameroon's Ministry of Public Health, Mvila District Health Management, WHO-AFRO, and international partners to translate these model-based projections into funded, pre-positioned epidemic preparedness action for Ebolowa before the next outbreak renders the arithmetic of delay irreversible.

## Data Availability

Publicly available datasets were analyzed in this study. This data can be found here: all WHO surveillance data used in this study are freely accessible at https://data.who.int and https://www.afro.who.int. The numerical simulation code is available from the corresponding author upon reasonable request.
